# 30 Years on Selected Issues in the Prevention of HIV among Persons Who Inject Drugs

**DOI:** 10.1155/2013/346372

**Published:** 2013-06-12

**Authors:** D. C. Des Jarlais, S. Pinkerton, H. Hagan, V. Guardino, J. Feelemyer, H. Cooper, A. Hatzatkis, A. Uuskula

**Affiliations:** ^1^The Baron Edmond de Rothschild Chemical Dependency Institute, Beth Israel Medical Center, 160 Water Street, FL 24, New York, NY 10038, USA; ^2^Department of Psychiatry and Behavioral Medicine, Center for AIDS Intervention Research, Medical College of Wisconsin, Milwaukee, WI 53226, USA; ^3^NYU College of Nursing, New York, NY 10003, USA; ^4^Rollins School of Public Health, Emory University, Atlanta, GA 30329, USA; ^5^Department of Hygiene, Epidemiology and Medical Statistics, Athens University Medical School, 11527 Athens, Greece; ^6^Department of Public Health, University of Tartu, 50411 Tartu, Estonia

## Abstract

After 30 years of extensive research on human immunodeficiency virus (HIV) among persons who inject drugs (PWID), we now have a good understanding of the critical issues involved. Following the discovery of HIV in 1981, epidemics among PWID were noted in many countries, and consensus recommendations for interventions for reducing injection related HIV transmission have been developed. While high-income countries have continued to develop and implement new Harm Reduction programs, most low-/middle-income countries have implemented Harm Reduction at very low levels. Modeling of combined prevention programming including needle exchange (NSP) and antiretroviral therapy (ARV) suggests that NSP be given the highest priority. Future HIV prevention programming should continue to provide Harm Reduction programs for PWID coupled with interventions aimed at reducing sexual transmission. As HIV continues to spread in low- and middle-income countries, it is important to achieve and maintain high coverage of Harm Reduction programs in these locations. As PWID almost always experience multiple health problems, it will be important to address these multiple problems within a comprehensive approach grounded in a human rights perspective.

## 1. Introduction

We now have three decades of experience in HIV prevention for persons who inject drugs (PWID); a vast amount of data has been collected, and much is known. In this paper we will briefly review what has been learned in these three decades and discuss what we believe are several critical issues for future research and public health practice with respect to HIV and injecting drug use. We will not, however, undertake a systematic review of the epidemiology of HIV infection among PWID nor a review of the implementation of various interventions to prevent HIV infection among PWID. For those topics, we would refer readers to the Lancet series [1] and the most recent UNAIDS Annual Report [2]. (Though we would note much of this epidemiological and service provision information needs to be updated.)

We will also not examine in depth the current international economics of HIV prevention for PWID. We would note that due to the international economic recession, there is declining international support for HIV prevention for PWID. The global fund for AIDS, tuberculosis, and malaria has had considerable difficulties in raising monies from high-income countries [3, 4], and the US President's Emergency Program for AIDS Relief (PEPFAR), the largest single donor program, is now moving towards a “country ownership” stage, in which the national governments in PEPFAR recipient countries will be expected to contribute more financial resources and assume more managerial responsibility for HIV prevention and treatment in their countries. There is always the danger that programs for socially marginalized groups such as PWID will disproportionately suffer from funding reductions. 

Before discussing the selected topics, however, it will be useful to consider the historical circumstances under which research has been conducted on HIV and injecting drug use. The context in which the research has been conducted is in some ways as important as the actual outcomes of the research. 

## 2. Initial Historical Contexts

When the first cases of what is now called AIDS were identified among drug injectors in 1981 [5], it was clear that it was a fearsome disease, but it seemed modest in scale. There was a concentration of cases in New York City and only scattered cases throughout the rest of the USA and Western Europe. The discovery of HIV as the causative agent for AIDS and the development of the antibody test for HIV dramatically changed the scale of the problem. More than half of drug injectors in New York City [6] and Edinburgh, Scotland [7], were infected as were a third of injectors in Amsterdam [8].

The realization of the scale of the problem generated a sense of great urgency in some public officials. It was clear that the virus was transmitted through multiperson use (sharing) of needles and syringes used for injecting drugs, so that programs which might reduce muti-person use were needed immediately. For example, the Netherlands initiated the first syringe exchange program in 1984 that was quickly expanded when HIV infection was noted among PWID there [9]. The United Kingdom followed and quickly began a pilot program for syringe exchange and following evaluation implemented a national syringe exchange program [10]. Australia [11] was also quick to implement national syringe exchange programs for PWID.

The HIV epidemic among drug injectors also became enmeshed in the politics of illicit drug use, particularly in the USA. At the time, the USA was experiencing a crack cocaine epidemic [12] that included considerable public violence related to the distribution of the drug. This fed into the concern that nothing should be done that might “encourage” drug use. Racial tensions also fed into the concerns about HIV prevention, particularly syringe exchange, with initial intense opposition from African-Americans in New York City [13].

Neither the urgency of implementation in areas where there was the political will to implement strong HIV prevention programs nor the fear of possible adverse consequences of prevention programs where the political will did not exist was conducive to methodologically rigorous research. In the places where there was the political will to provide controversial prevention programs, research typically involved conducting pilot studies followed by large-scale implementation. Very rigorous research, such as randomized controlled trials, was not considered necessary or ethical. In the USA, which has historically contributed the greatest amount of funding for drug use research, federal policy not only prohibited using federal funds to provide syringe exchange services but also prohibited using federal funds to even conduct research on syringe exchange programs. Private foundations fund syringe exchange research in the USA, and as a result only a very modest amount of resources is available for this research. 

There is also very great complexity in epidemics of injecting drug use, epidemics of HIV among PWID, and interventions to reduce HIV transmission among PWID. Both epidemics of drug injecting and of HIV may change over time, often quite rapidly. When prevention programs are brought to scale, they become complex organizational phenomena, particularly if different types of interventions are implemented at the same time (combined prevention programming). Thus, there are inherent limits to the precision of our knowledge about the successes (and occasional failures) of interventions to reduce HIV transmission among persons who inject drugs. Although imprecision exists, there is now a sufficient evidence base for a consensus of recommendations regarding the types and scale of interventions that should be implemented for the prevention and care of HIV among PWID [14].

First, we know that injecting drug use and HIV infection among PWID continue to spread globally. In 2004 there were 130 countries with injecting drug use and 78 countries with HIV among PWID [15]. In 2008 there were 148 countries with injecting drug use and 120 countries with HIV among PWID [1]. The same factors that have led to great increases in global trade overall—improvements in transportation, communication, fewer restrictions on the flow of capital—have also led to increased trade in illicit drugs. Given these factors and the tremendous profits to be made in the distribution of illicit drugs, there would not appear to be any immediate likelihood of reducing the illicit supply of psychoactive drugs. We will therefore need to continually address the many health problems associated with injecting drug use.

Second, we know that HIV can spread very rapidly among PWID with increases in HIV prevalence from 10% to 50% per year [16]. Thus, a situation in which HIV does not appear to be a threat among PWID can rapidly change to a situation in which a high seroprevalence epidemic has already occurred. 

Third, we know that it is possible to avert HIV epidemics among PWID. Large-scale implementation of HIV prevention programs, particularly needle/syringe access programs, when HIV prevalence is very low in a population of PWID can keep the prevalence low (under 5%) indefinitely [10, 11, 17, 18]. It is important to note, however, that there have been instances of outbreaks of HIV when it appeared that HIV was under control in the local PWID population. The most famous of these is probably the Vancouver outbreak in the mid-1990s [19], and the most recent outbreak of HIV among PWID that occurred in Greece [20]. 

In Vancouver, the outbreak appears to have been generated by a concentration of social/economic problems in the Downtown Eastside area and a change from primarily heroin to primarily cocaine injection. As cocaine may be injected much more frequently than heroin and the local syringe exchange program had a strict limit on numbers of syringes that could be obtained per week, the change to cocaine injecting would have generated many more injections per syringe distributed by the exchange program. In Greece and Romania, HIV had remained low despite inadequate prevention programs, but changes in the economic situation led to increased economic disparities followed by increased injecting risk behaviors followed by the HIV outbreak [21]. A detailed study of the outbreak in Greece is currently underway, and the preliminary findings show a complex series of events that led to the outbreak [20, 22]. Beginning in 2007, the Greek economy entered into a severe recession, which led to a reduction in public services and increased homelessness. Low coverage of prevention services, homelessness and economic disparities provided opportunities for multiperson use of needles and syringes with large numbers of other persons, and the supply of sterile needles and syringes was quite limited. Increased transmission of hepatitis C virus (HCV) was observed among PWID in Athens, prior to the outbreak of HIV. The stage was being set well before the actual increase in HIV transmission. 

Phylogenetic analyses of the different strains of HIV in the outbreak show at least four separate outbreaks, two of which appear to have been strains already existing among PWID in Athens and two of which appear to have been introduced by international travel among PWID. A strong public health response has been initiated with increased syringe distribution, HIV counseling and testing, increased methadone treatment, and increased antiretroviral treatment for HIV seropositive PWID in Athens. It is hoped that this response will quickly bring the HIV outbreak under control. 

Vancouver and Greece thus serve as examples of areas where HIV appeared to be under control, but where economic conditions and/or drug injection patterns changed rapidly and, despite the existence of some prevention programs, major outbreaks of HIV then occurred.

Fourth, we know that it is possible to “reverse” high HIV seroprevalence epidemics among PWID. Very large declines in HIV incidence have been observed after large-scale implementation of evidence-based prevention programs, particularly when multiple prevention programs (needle/syringe programs, substance use treatment programs, HIV testing, and antiretroviral treatment) are implemented simultaneously [14, 23]. Examples include Amsterdam [9], Australia [18], Italy [24], New York City [25], Scotland [7], Spain [26] and Vancouver [27].

While much has been learned in 30 years of research on reducing HIV transmission among PWID, there are still a number of critical issues that need to be addressed. This paper will discuss what we believe are several of the most important current issues. From the previous discussion, it is clear that one of the most important considerations is the policy context within which HIV epidemics have (or have not) occurred among PWID. This will be addressed at the conclusion of the paper. 

## 3. Doing HIV Prevention for PWID in Resource-Limited Settings

The previous examples of highly successful HIV prevention programming are all from high-income countries. Most—but certainly not all—of current HIV transmission is occurring in low- and middle-income countries [1], and we do not yet have sufficient long-term data from HIV prevention programming in resource-limited settings to draw any firm conclusions with respect to effectiveness. There are multiple reasons for the lack of long-term data on the effectiveness of HIV prevention for PWID in low- and middle-income countries; HIV epidemics among PWID in low- and middle-income countries generally occurred more recently than HIV epidemics among PWID in high-income countries, implementation of HIV prevention in low- and middle-income countries is generally at very low levels [28], and there have generally been insufficient resources for conducting long-term outcome studies.

There are several concerns for why prevention programming may not be as effective in low- and middle-income countries as in high-income countries. First, there is the simple scarcity of resources for prevention programs. Some types of HIV prevention programs, particularly long-term drug treatment programs and antiretroviral treatment for HIV, are comparatively expensive, and it may not be possible to provide these on a public health scale in many low- and middle-income countries. This is not simply a matter of financial resources but also a matter of appropriately trained health workers.

Second, while PWID are stigmatized in almost all countries, the stigmatization may be particularly severe in many low- and middle-income countries. This may lead political leaders to be less willing to allocate resources to HIV prevention and treatment for PWID. In particular, to the extent that injecting drug use is seen as a practice associated with degenerate Western culture, nationalism in low- and middle-income countries may lead political leaders to fail to implement evidence-based HIV prevention programs for PWID [29, 30]. The stigmatization of injecting drugs may compound the stigmatization of having (or simply being at risk for) HIV, leading PWID to avoid using the programs that are available.

Third, effective HIV prevention programs require at least passive cooperation from law enforcement, and relationships between drug users and law enforcement may be particularly problematic in many low- and middle-income countries. In some countries, drug addiction may be a status offense (simply being an addict is sufficient basis for incarceration, without having to be found in possession of drugs). Many low- and middle-income countries also have official registries of persons known to be addicted, and persons on these registries may lose important civil rights. Addicts may also be subject to police brutality [31, 32]. Thus, drug users may be very reluctant to participate in HIV prevention activities if such participation risks exposure to law enforcement. Carrying clean needles and syringes, in particular, may be risky for drug users in such settings.

 Finally, and of critical importance, political leaders in some transitional, low- and middle-income countries have viewed injecting drug use as a foreign, “decadent” behavior from the West that must be resisted in order to protect cultural traditions. Thus, interventions that appear to accept continuing drug use (such as syringe exchange) or appear to merely substitute the use of one narcotic drug for another (methadone maintenance treatment) are strongly resisted regardless of any scientific evidence. One of the most important examples of this cultural resistance is the Russian opposition to methadone maintenance treatment [33, 34].

## 4. Modeling

In the relative absence of high quality, long-term data on the effectiveness of combined prevention programming for PWID in low- and middle-income countries, modeling of the effects of combined prevention programming may be particularly useful for allocation of the scarce resources. We have conducted modeling for HIV prevention among PWID in Estonia, a small Baltic country that was formerly part of the Soviet Union. Similar to many other newly independent countries, with the dissolution of the Soviet Union, Estonia experienced epidemics of sexually transmitted infections, injecting drug use, and HIV among drug injectors [35, 36]. Estonia recorded the highest per capita rate of HIV infections of any country in Eastern Europe.

The model that we used to estimate the effectiveness of syringe exchange and ART in reducing annual HIV incidence among PWID in Estonia is described in Pinkerton [37]. This model is based on Edward Kaplan's original needles that kill model [38]. To include the effects of antiretroviral treatment (ART) in reducing HIV incidence, we assumed that half of the persons on ART had reached viral suppression and thus were no longer capable of transmitting HIV through injection or sexual risk behavior. This has the effect of reducing HIV prevalence by the number of persons who have reached viral suppression. 

In this model, the incidence of new HIV infections in a particular population of PWID is a direct function of the rate of injections with borrowed syringes and the proportion of borrowed syringes that are contaminated with HIV. In symbols,

(1) $$\begin{array}{c} \text{incident}\;\text{infection}\;\text{rate}=\left(1-p\right)lca, \end{array}$$

where *p* is the prevalence of existing infection in the PWID community (hence 1 − *p* is the probability that a particular PWID is at risk of infection), *l* is the average number of injections with borrowed syringes per PWID per unit time, *c* is the proportion of borrowed syringes that are contaminated with HIV, and *a* denotes the per-injection probability of HIV transmission from a contaminated syringe to a previously uninfected PWID. The contamination rate, *c*, is determined by another equation (not shown) that critically depends upon the rate at which used syringes are exchanged for sterile ones—the greater the exchange rate, the shorter the average time each syringe spends in circulation, hence the less likely it is to become contaminated with HIV.

 To include the incremental impact of antiretroviral therapy (ART) on HIV transmission among PWID, we assumed that 50% of PWID on ART achieve viral suppression and are no longer capable of contaminating a shared syringe. This directly reduces the likelihood that a shared syringe will become contaminated with HIV.

**Figure 1:**
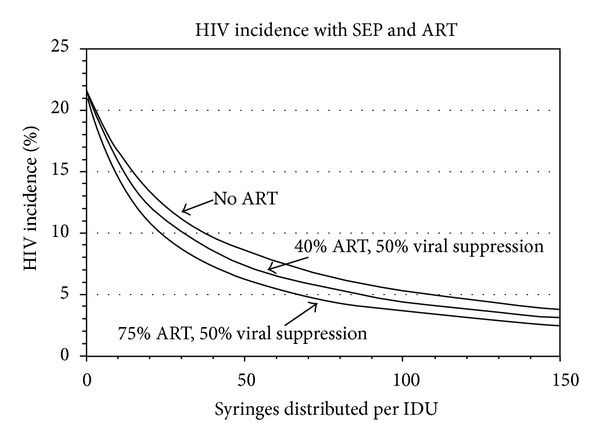
Modeling of effects of syringes distributed and ART and on annual HIV incidence among PWID in Tallinn, Estonia. The impact on incidence of SEP combined with 2 levels of ART coverage (40% and 75%), assuming 50% of those on ART cannot contaminate a syringe.

We modeled the combined impact of syringe exchange and provision of ART for PWID in Tallinn, Estonia.  Figure 1 shows the results of the modeling. The individual curves show the relationships between the numbers of sterile syringes distributed per PWID per year and HIV incidence. All of these curves show large reductions in HIV incidence as the numbers of syringes distributed increase from low to moderate levels. All of the curves do flatten, however, with less absolute reduction in HIV incidence as the numbers of syringes distributed per PWID increase from high to very high levels. The different curves represent the relationship of the effects of providing ART to HIV seropositive PWID in a population with 50% HIV prevalence (as currently in Tallinn). The top curve represents no ART, the middle curve represents providing ART to 40% of seropositives, and the bottom curve represents providing ART to 75% of the HIV seropositives in the PWID population (again, with the assumption that half of those receiving ART have reached viral suppression and are no longer infectious).

Opiate substitution treatment (OST) was not included in this model. Because the relatively high cost of OST in Estonia has limited the number of OST-methadone treatment positions to such a modest number (approximately 200 in Tallinn for an injecting population of approximately 6000). OST is not likely to have any noticeable effect on the course of the epidemic. If one assumes that OST effectively keeps patients from injecting illicit drugs, then OST could readily be incorporated into the model by reducing the injecting population by the number of persons currently receiving OST. 

This model does suggest that the highest priority be given to expanding needle/syringe programs as the first intervention for reducing HIV transmission among PWID. Needle/syringe programs can reach high coverage at relatively low cost and do not require the expensive, highly trained staff needed (physicians, nurses, and pharmacists) for ART and opiate substitution programs. 

A model proposed by Strathdee and colleagues has also been used to assess combined HIV prevention programs for PWID [39]. It is interesting to compare the modeling of combined HIV prevention programs for PWID in Estonia with the modeling proposed by Strathdee and colleagues for combined prevention programming for PWID in Ukraine, another Eastern European country with an IDU-concentrated epidemic. The model proposed by Strathdee et al. was based on the effectiveness of combined prevention programming in the Amsterdam Cohort Study [9]. Strathdee and colleagues concluded that combined syringe exchange, ART, and OST (opiate substitution therapy, methadone and/or buprenorphine maintenance) with 50% uptake of each intervention would reduce HIV incidence among PWID in Ukraine by 41% with a confidence interval of 18% to 63%.

The two models both indicate that combined prevention programming would lead to very substantial reductions in HIV incidence among PWID in the two countries, though the Pinkerton-Estonia model suggests a greater reduction in HIV incidence.

Neither model, however, indicates anything close to elimination of HIV transmission—with incidence rates <0.5/100 person-years as currently observed in places like Amsterdam [23], New York City [25], or Vancouver [27]. Determining the minimum HIV incidence that can be achieved through combined HIV prevention for PWID in low- and middle-income countries is clearly an area in which additional empirical research is needed.

Cost factors also need to be included in modeling efforts for low-middle-income countries, as it is important to allocate scarce resources to interventions that are likely to have large effects.

## 5. After Success Then What? The Future of HIV Prevention Programs after the Threat of AIDS Has Been Greatly Reduced (in Some Countries)

With the very real success of HIV prevention for PWID in many high-income countries, the trends to reduce funding for public health activities among many governments throughout the world, the lack of a strong political constituency for PWID, and continuing ideological opposition to some of the most effective prevention programs, there are now pressures to reduce funding for HIV prevention programs among PWID. Even in areas where overall HIV prevention funding is not being reduced, there is pressure to reallocate funding from prevention among PWID to prevention among groups in which HIV incidence is increasing (notably men-who-have-sex-with-men) [40].

There are several epidemiological and economic reasons why HIV prevention programs should not be drastically reduced even if injecting-related transmission of HIV is near zero in a local PWID population. First, there is always the likelihood of sexual transmission leading to new HIV infections among PWID. Sexual transmission may occur from a person who does not inject drugs to a person who does inject, or a person who does not inject may acquire HIV sexually and then begin to inject drugs. Second, travel by PWID from other areas may reintroduce HIV into the local population, with the threat of new outbreaks, or a member of the local PWID population may travel, acquire HIV, and then return to the local population. Third, drug users may cycle through periods of less versus more frequent drug use and of less to more frequent HIV risk behaviors, so that what was a very low risk situation may change fairly rapidly. Population level drug use patterns may also change fairly rapidly. The introduction of cocaine injection in several Canadian cities was followed by increases in HIV incidence [41, 42]. In the absence of effective prevention programs for PWID, any of these might initiate an outbreak of HIV in the local population.

If an outbreak of HIV was to occur in an unprotected PWID population, it could be very costly in terms of health and finances. The most recent estimate of the cost of medical treatment for a single HIV infection in the USA was estimated to be $367,134 in 2009 dollars and $379,668 in 2010 dollars [43]. The most recent cost estimate for HIV infection in Germany was over 23,000 euros per year [44]. Thus, even a modest number of new cases of HIV would cost much more than the savings from reducing HIV prevention programs.

With their success in reducing HIV transmission, HIV prevention programs have evolved beyond their original purpose. In the USA, many of them have become frontline multiservice programs for PWID, providing not only sterile injection equipment and condoms but also HIV counseling and testing, referrals to drug treatment, hepatitis C counseling and testing, hepatitis A and hepatitis B vaccination, and distribution of naloxone for reversing opiate overdoses among other services.  Table 1 shows various services provided by the US syringe exchange programs in 2011 (data from [45]). In Western Europe, syringe exchange programs are becoming more integrated with other health and social services for PWID [46].

Drug treatment programs have also been evolving beyond their original purpose towards providing additional services, including HIV testing, vaccination for hepatitis B and C, and on-site primary medical care [47]. As the population of drug users ages in the USA, drug treatment programs also have to address geriatric issues [48]. Some drug treatment programs are also providing directly observed HIV antiretroviral therapy (ARV) [3, 49, 50].

Prevention and treatment of hepatitis C virus (HCV) infection may be among the most important new goals for both syringe exchange and drug treatment programs. HCV is much easier to transmit through multiperson use of injection equipment than HIV and is hyperepidemic in most populations of PWID [51–53]. HCV is already likely to cause more deaths among PWID than HIV does in the USA [54, 55] and in other high-income countries.

The expansion of services at both syringe exchange and drug treatment programs raises the issue of coordination between these historically different types of services. Integration of services for PWID is still an ongoing process, however, and the stigmatization of PWID remains an enduring problem. There continues to be high levels of imprisonment for drug use in many countries, although in recent years there has been a progression towards decriminalizing use in favor of drug treatment through recommendations from the Global Commission and specific UN agencies [56].

## 6. Sexual Transmission of HIV among PWID and from PWID to Nondrug Injecting Partners 

Many persons who inject drugs are sexually active, so HIV infection among PWID raises the possibility of HIV transmission to sexual partners who do not inject drugs and of an HIV injecting drug use concentrated epidemic leading to a heterosexual HIV epidemic. This is, of course, a difficult question on which to conduct research, as studies need to be conducted at a population level and the potential causal lag periods need to be examined carefully. The first international systematic review of possible transitions from IDU concentrated to heterosexual epidemics found that the most important factor in preventing such transitions is having a short period of high HIV incidence among PWID [16]. While additional research is clearly needed on this topic, these first findings provide strong additional rationale for scaling up HIV prevention programming for PWID as early as possible.

There is also the problem of sexual transmission of HIV among persons who inject drugs and from persons who do not inject to persons who do inject. As injecting-related transmission is brought under control, sexual transmission among and to persons who inject becomes of greater importance, most likely accounting for the majority of new HIV infections among PWID in some locations [57–59].

Condom distribution and psychosocial/education interventions that reduce sexual risk behavior are still the most commonly used interventions to reduce sexual transmission of HIV among, from and to PWID. These programs do have meaningful effects in reducing sexual risk behavior [60], but they are not nearly as effective as needle/syringe access programs. PWID are much more likely to consistently use clean needles and syringes than condoms.

Treatment as prevention [61, 62], in which HIV seropositive PWID are given antiretroviral therapy in order to reduce their HIV viral load and therefore their likelihood of transmitting HIV, is a promising new strategy for reducing sexual transmission among PWID. Such a strategy may become quite effective in high resource settings, but there are difficult resource and logistical issues that would need to be overcome in resource-constrained settings. For example, a national study conducted in China among serodiscordant couples conducted between 2003 and 2011 found that antiretroviral therapy was effective in reducing HIV transmission among infected couples in all at risk groups except for those in which at least one of the individuals injected drugs [63].

## 7. Policy Issues/Structural Determinants of Continued HIV Transmission

After 30 years of research on HIV transmission among persons who inject drugs, we should be in a position to create an “AIDS free generation” [64]. While HIV is readily transmitted through the multiperson use of injecting equipment, when the means for safer behavior are available, drug users have been remarkably adept in reducing their injecting risk behavior. As noted previously, there are many areas in which HIV epidemics have been averted among PWID and many areas in which high HIV prevalence epidemics have been brought under control, with close to zero new infections. However, also as noted previously, HIV continues to spread among PWID in many areas of the world. In part, this is due to the limited resources available in many low and middle-income countries, but drug policy issues are equal, if not more important. Implementation of effective HIV prevention interventions requires an appropriate policy framework.

At the risk of some oversimplification, it is possible to discern two diametrically opposed policy approaches to psychoactive drug use. One perspective may be termed a “War on Drugs.” Within this perspective, the use of illicit drugs is considered to be immoral behavior that is best controlled through criminal punishment. These punishments are to be applied to both persons who distribute and who use the drugs. There are several important factors in this perspective: first, illicit drug use if often associated with a perceived corrupting influence of “foreign/outsider” groups. For example, opium use in the USA and Australia was associated with Chinese immigrants [65], cocaine use was associated with African-Americans [66], and marijuana use with Mexican-Americans [67]. More recently, injecting drug use is often seen in transitional and low- and middle-income countries as a corrupting influence of Western societies. In Russia, the introduction of heroin from Afghanistan is often seen as a plot by Western countries to undermine traditional Russian culture [68]. Thus, opposition to injecting drug use then becomes a defense of traditional moral values, in which the symbolism of actions in opposition to drug use may be more important than the practical consequences of the actions. 

Second, within a War on Drugs perspective, any actions that might appear to “encourage” or “condone” drug use must be resisted. Such actions—providing sterile injection equipment in particular—threaten the integrated cohesive framework of the War on Drugs. Deviation from the “War” perspective is seen as a slippery slope leading to “legalization” of drugs.

Third, this perspective is often immune to evidence. Data indicating that a particular aspect of an approach is not working is not seen as a reason for reevaluating the approach but rather as a reason for increasing the resources used for applying the approach. The increase in the length of prison sentence for drug possession (the “Rockefeller” laws in New York State) is an example where criminal punishments were not working which was followed by increases in the criminal punishments [69]. 

Finally, the approach of relying on law enforcement to control psychoactive drug use originated around the turn of the twentieth century [70], before there was much scientific knowledge about the effects of different psychoactive drugs (including drugs that are currently legal), well before the globalization of the trade in licit and illicit psychoactive drugs, and well before the widespread transmission of blood-borne viruses (HIV and hepatitis C) through multiperson use of injecting equipment and drug use influenced unsafe sexual behavior. These historical conditions, however, have clearly changed.

The contrast to a War on Drugs perspective is often called a “Harm Reduction” perspective [71] or more generally a public health perspective. While there are many variations on this perspective, there are several essential components of it. First, a Harm Reduction perspective is pragmatic. Policies and practices to address substance use should be based on the best available evidence. The pragmatism includes addressing drug use-related problems that may be ameliorated without necessarily reducing the drug use itself. Reducing HIV transmission among persons who continue to inject drugs may be the clearest example of reducing a serious adverse consequence of drug use without necessarily reducing the drug use itself. Distributing naloxone to reverse opiate overdoses is another example of reducing a drug-related harm without necessarily reducing the underlying drug use. 

An important aspect of the pragmatism of a Harm Reduction perspective is realization of the limits of our current knowledge about substance use disorders. While there are effective treatments to manage substance use disorders, we do not have any simple “cures” for these disorders. Similarly, while there are programs that reduce substance use among youth [72], these programs have not been able to prevent periodic increases in the use of different drugs [73], nor were any prevention programs able to prevent the international increases in methamphetamine use over the last decade [74]. 

The second major component of harm reduction is an emphasis on human rights. As noted in the Vienna Declaration [75], the human rights of drug users are frequently violated in many countries. With respect to HIV, the failure to provide evidence-based prevention and care for a potentially fatal disease is considered to be a fundamental violation of human rights. There is increasing international recognition of a “right to health” [76], and while this right may be aspirational rather than fully implemented in many countries, the failure to provide HIV prevention and care for PWID is a clear example of violation of human rights. 

The “detention centers” operated in some countries in Asia are a particularly egregious violation of the human rights of PWID. Drug users may be imprisoned in such centers without any due process, be subject to forced labor, and rarely receive any evidence-based treatment. Drug users traditionally have been incarcerated in these centers under the rationale that this would lead them to stop using drugs, but relapse rates are quite high (typically 90% or greater) according to government statistics [77].

Drug users may also be subject to police brutality [26, 27]. The possibility of being detained in a center or subject to police brutality may lead many drug users to avoid using HIV prevention and care services that are available.

Promoting the Harm Reduction/public health/human rights perspective on drug use may require reducing the stigmatization of drug users and of persons with (or at risk for) HIV. There have been a modest number of studies of interventions to reduce stigmatization related to illicit drug use [55] and to HIV [56], but these studies generally suffer from methodological weaknesses and have not shown large effect sizes. Much more systematic research is clearly needed in this area. 

## 8. Summary/Conclusions

In 30 years of research on and implementation of HIV prevention for PWID, there have been some remarkable successes. In high-income countries, the reduction of injecting-related HIV transmission has been second only to the reduction in mother to child transmission. HIV is still spreading in many low- and middle-income countries, however, and there are good reasons to expect that both injecting drug use and HIV among PWID will continue to spread to additional countries. Lack of resources is a major problem for HIV prevention in low- and middle-income countries, but stigmatization of drug users and lack of political will to implement evidence-based programs are probably the biggest problems.

The successes in HIV prevention have led to a broadening of goals for improving the health of PWID. This broader vision of addressing health needs among PWID will need to be based on a human rights perspective. A human rights basis is necessary for addressing the multiple health issues of PWID. There are strong economic arguments to be made for preventing HIV infection among PWID—the costs of treating HIV infection are much greater than the costs of preventing infection—but a comprehensive approach to addressing health problems among PWID needs to be grounded in human rights rather than just in economics. 

## Figures and Tables

**Table 1:** Services provided at United States Syringe Exchange Programs, 2011.

Service provided by SEPs in 2011	Percentage of SEPs offering service
HIV counseling and testing	81%
Hepatitis C education and counseling	85%
Hepatitis C testing	62%
Hepatitis C treatment	4%
Hepatitis B education and counseling	69%
Hepatitis B testing	18%
Hepatitis B vaccine	42%
Hepatitis A education and counseling	66%
Hepatitis A testing	12%
Hepatitis A vaccine	40%
Distribution of naloxone	47%
Substance abuse treatment referrals	94%
STD screening	47%
Tuberculosis screening	26%
